# Scaling Diradicaloid Thermoelectricity

**DOI:** 10.1021/acs.nanolett.6c03798

**Published:** 2026-09-25

**Authors:** Lewis Hamilton, Jiung Jang, Amit Sil, Peng He, Abdalghani H. S. Daaoub, Yongjun Choi, Sara Sangtarash, Andrea Vezzoli, Hyo Jae Yoon, Hatef Sadeghi

**Affiliations:** † Quantum Device Modelling Group, School of Engineering, 2707 University of Warwick, Coventry CV4 7AL, U.K.; ‡ Department of Chemistry, 34973 Korea University, Seoul 02841, Republic of Korea; § Department of Chemistry, 4591 University of Liverpool, Crown Street, Liverpool L69 7ZD, U.K.; ∥ Department of Chemical Sciences, Bose Institute, Kolkata, West Bengal 700091, India

**Keywords:** Molecular thermoelectricity, Seebeck coefficient, Electrical conductance, Diradicaloids, bis(indeno)acene, Quantum transport, Self-assembled monolayers

## Abstract

The identification of molecular materials with high thermoelectric performance has attracted considerable attention due to their unique quantum properties, elemental abundance, and environmental friendliness. Achieving this requires overcoming the conventional tradeoff between the Seebeck coefficient (*S*) and electrical conductance (*G*). Here, we exploit the thermoelectric properties of diradicaloid molecular junctions and demonstrate the coenhancement of *G* and *S*. Calculations predict that elongating the diradicaloid backbone systematically reduces the energy gap, pushing transport resonances closer to the electrode’s Fermi energy, leading to the simultaneous improvement of *G* and *S*, and a length-dependent increase in power factor unlike that of conventional conjugated molecules. This is confirmed in self-assembled monolayers (SAMs) formed from diradicaloids, where we observe an increase in *G* with the diradicaloid length and *S* reaching values exceeding 40 μV/K. This shows exotic quantum transport properties of single-molecule diradicaloids translate to technologically relevant SAMs for next-generation high-performance thermoelectric energy conversion devices.

## Graphical abstract

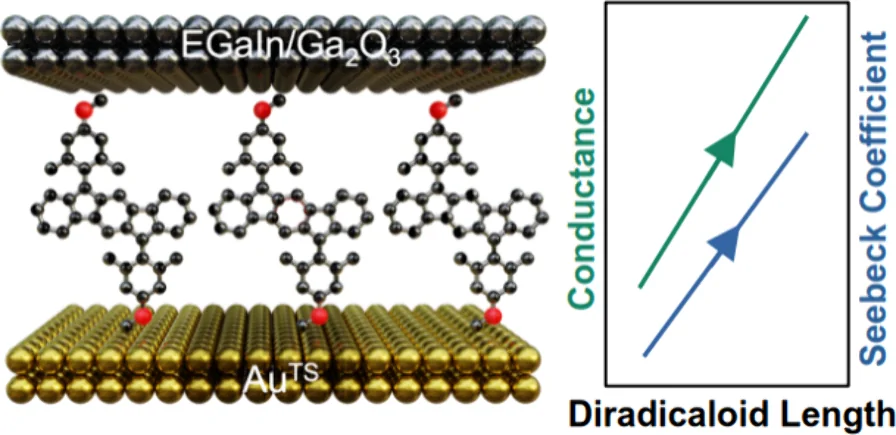

Thermoelectricity, the conversion of thermal energy into electrical energy, offers highly attractive solutions to modern energy challenges, ranging from waste heat harvesting and wearable devices to Peltier refrigeration.^1^ Identifying materials with high thermoelectric performance remains a challenge in molecular electronics.^2–6^ Organic materials formed primarily of π-conjugated molecular systems are particularly promising candidates.^7^ These organic materials typically offer low phonon thermal conductance (*κ*_*p*_) due to their lower mass and weaker intermolecular interactions.^8–13^ This suppression of the phonon contribution to thermal conductance in turn can yield higher values for the overall thermoelectric figure of merit, *ZT* = *P* *T*/κ where *P* is the power factor, *T* the temperature, and *κ = κ_e_ + κ_p_* the thermal conductance due to electrons (κ_*e*_) and phonons (κ_*p*_). In order to increase *ZT*, and therefore enhance efficiency of a thermoelectric device to convert heat to electricity, the power factor *P* = *GS* ^2^ also needs to be improved. However, in typical thermoelectric materials, attempting to increase *S* is often accompanied by a reduction in *G*.^14,15^

In the pursuit of overcoming this trade-off, molecular materials offer unique opportunities because they can exhibit sharp transport features close to the energy of molecular frontier orbitals. These sharp features are essential because *G* is proportional to the amplitude of the transmission coefficient *T*(*E*) at *E*_F_, while *S* is proportional to its slope (*d* ln­(*T*(*E*))/d*E*); thus, sharp resonances near *E*_F_ can simultaneously lead to high-*G* and high-*S*.^16–18^ In most conventional molecules, however, transport resonances are not located close enough to *E*_F_ to realize this coenhancement. Consequently, the field has increasingly turned its attention toward organic molecular materials with open-shell properties, such as Blatter radicals, which show immense potential.^17^

Open-shell derivatives with partially filled orbitals can lead to new transport resonances, favorable transitions from destructive to constructive quantum interference, and significantly smaller energy gaps between frontier orbitals.^17–21^ These features collectively contribute to higher conductance and closer orbital alignment to the Fermi level of the electrodes, fundamentally altering the slope and value of *T*(*E*) to enhance both *G* and *S*. Despite this potential, leveraging these materials is practically challenging because radicals that remain stable at room temperature are currently limited to exceptional cases such as Blatter, Nitroxide (TEMPO), Trityl, and Verdazyl radicals.^17–22^

Recently, we designed a series of quinoidal polycyclic hydrocarbons^21^ featuring a bis­(indeno)­acene core (Figure 1a) and established anti-Ohmic transport at the single-molecule level, with conductance increasing as the diradicaloid backbone is extended. These highly tailorable molecules exist with a sterically protected diradicaloid electronic configuration through the 3,5-dimethylthioanisolyl anchor groups to contact to the electrodes. A diradicaloid electronic configuration describes a partial diradical character characterized by resonances between a closed-shell configuration and a pure diradical form, achieved through the delocalization of unpaired electrons over the organic conjugated π-system. This configuration greatly enhances stability at room temperature while successfully retaining the highly attractive electronic properties of traditional radicals leading to a remarkable negative beta factor, a proportional increase in conductance as the molecular backbone length increased. These phenomena are associated with a significant reduction in the HOMO–LUMO gap and enhanced orbital alignment to the Fermi level. This is desirable for coenhancement of *G* and *S*, and therefore, in this paper, we investigate for the first time the thermoelectric properties of diradicaloids both theoretically and experimentally. Here, we address a distinct question: whether unusual electronic structure of diradicaloids can simultaneously enhance *G* and *S*, and whether these single-molecule trends persist in large-area self-assembled monolayers. We therefore combine quantum-transport calculations with ensemble thermovoltage and current-density measurements to establish the length dependence of *S*, *G*, and the thermoelectric power factor. We demonstrate that the exceptional single-molecule transport properties successfully translate into scalable SAM configurations (Figure 1b), yielding both simultaneous enhancement and high values of *G* and *S*.

**Figure 1:**
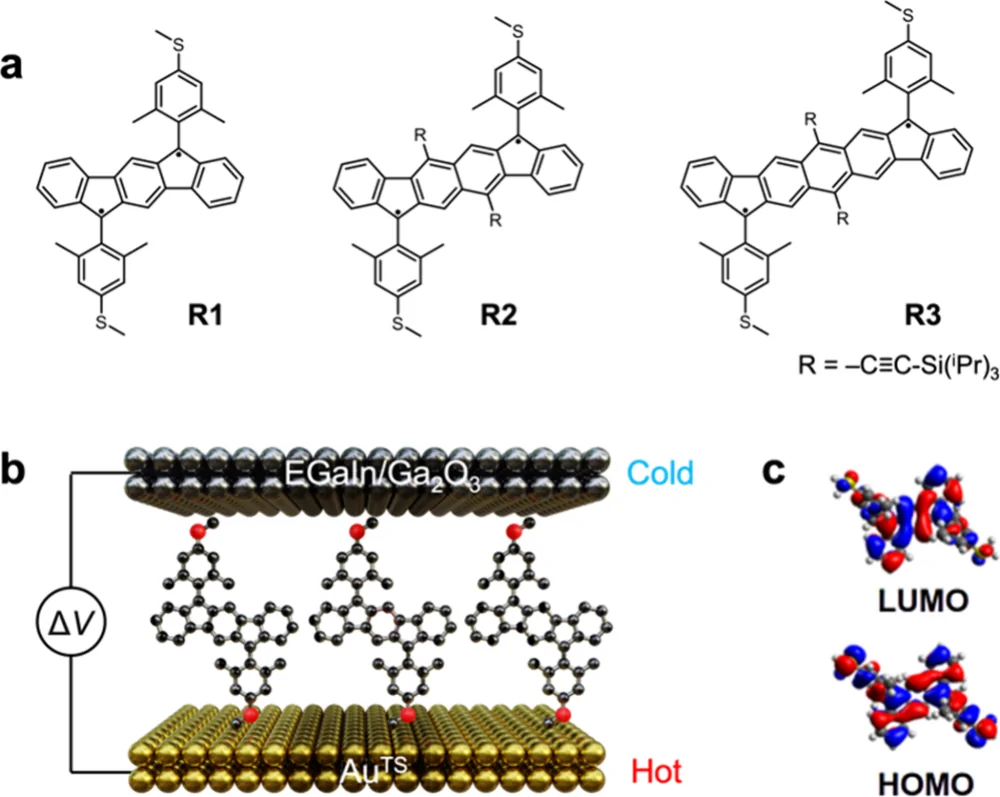
Diradicaloid molecular junctions. (a) molecular structure of bis­(indeno)­acenes with three different core acene lengths **R1**, **R2**, and **R3**. (b) Idealized depiction of self-assembled monolayers (SAMs) formed by **R1** diradicaloids between hot and cold electrodes. (c) HOMO and LUMO iso-surfaces of **R1**.

To establish the fundamental thermoelectric behavior of the molecular junctions formed from diradicaloids, we first investigated the gas phase electronic properties of **R1**-**R3** (as shown in Figures S1–S3 of the Supporting Information (SI)) using Density Functional Theory DFT (See computational method). The ground state spin configuration is a singlet, and the frontier orbitals are delocalized over the molecules as shown in Figure 1c and Figures S1–S3. We then placed these molecules between electrodes and evaluated their transport properties utilizing the SIESTA^23^ implementation of DFT combined with the GOLLUM code for quantum transport and thermoelectric calculations.^24,25^ In these calculations, we employ the DFT+Σ method^16^ to determine the position of resonances relative to the Fermi energy of electrodes (see the Computational Methods section). Figure 2a shows the transmission coefficients (*T*) of electrons with energy *E* passing from one electrode to the other through **R1**–**R3**. The open-shell character of these diradicaloids leads to a progressively reduced HOMO–LUMO gap and sharp transmission features close to the Fermi energy (Figure 2a). We then calculated the room-temperature electrical conductance and corresponding Seebeck coefficient.

**Figure 2:**
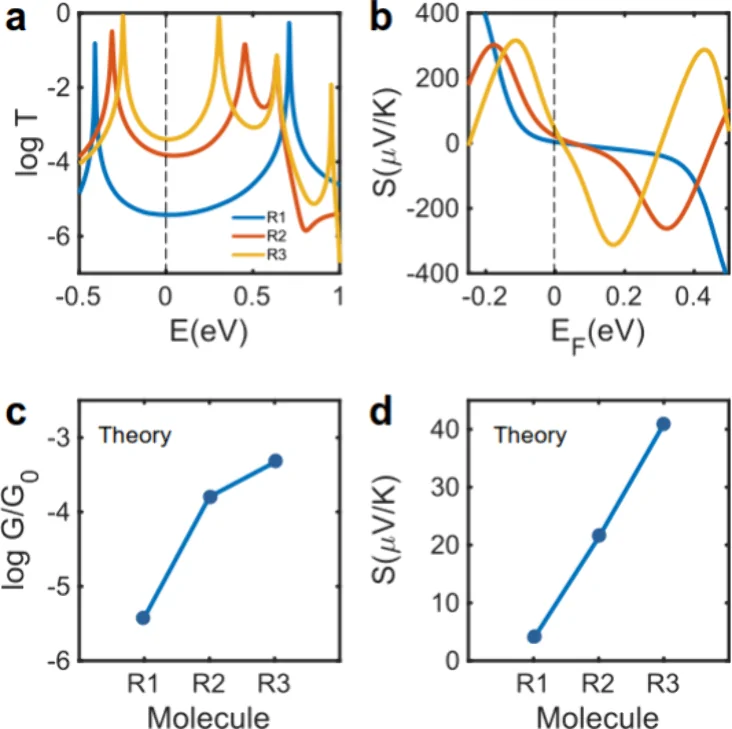
Calculated thermoelectric properties of bis­(indeno)­acene diradicaloids. (a) DFT+Σ transmission coefficient as a function of energy, (b) room-temperature Seebeck coefficient as a function of Fermi energy, (c) room-temperature electrical conductance, and (d) Seebeck coefficient as a function of length at DFT Fermi energy.

Figure 2b shows the Seebeck coefficient as a function of Fermi energy at room temperature. For all energies within the HOMO–LUMO gap of **R3**, the magnitude of *S* increases with molecular length (Figure 2b). This enhancement is driven by a rapid narrowing of the HOMO–LUMO gap in these diradicaloids with increasing length, which stems from a progressive increase in their diradicaloid character as the backbone length increases.^21^ This phenomenon can be rationalized through Clar’s rule, and the resulting trend in increasing aromatic stabilization as the number of π sextets also increases across the series **R1 → R3**. This narrowing brings the molecular frontier orbitals and associated resonances closer to the electrode’s Fermi energy, enhancing the Seebeck coefficient, as *S* is proportional to the slope of the transmission at the Fermi energy. This leads to remarkably high *S* for an organic molecular junction at room temperature, where values are typically much smaller and often below ±20 μV/K.^4,26^

This enhancement of the *S* by the backbone length leads to a positive Seebeck attenuation factor (Figure 2d). Furthermore, since the transport resonances get closer to the Fermi energy as a result of the reduced HOMO–LUMO gap by length, the amplitude of transmission probability at the Fermi energy increases leading to the enhancement of *G* (Figure 2c). This contrasts with the conventional thermoelectric tradeoff, where increasing *S* is often accompanied by reduced *G*. The significant coenhancement of *S* and *G* as shown in Figure 2c and 2d is key to enhancing the power factor, leading to values of 6.5 × 10^–5^ pW/K^2^ per molecule in **R3**, over an order of magnitude greater than OPE3, a widely used molecular-electronics benchmark.^27^

To confirm this and demonstrate that the enhanced thermoelectric properties can also translate to technologically relevant SAM junctions, we synthesized the **R1**–**R3** molecules and fabricated large-area SAMs. The partial open–shell character of **R1**–**R3** has been confirmed in our previous study^21^ by optical and magnetic measurements: visible spectroscopy shows significant absorption well into the near-infrared region, while variable-temperature NMR was used to characterize the small paramagnetic population that causes signal broadening. Furthermore, we performed thermally assisted occupation DFT calculations to estimate the degree of diradicaloid character *y*_0_ as defined by Yamaguchi.^28^ We found a clear trend of increasing *y*_0_ across the series, from *y*_0_ = 0.13 for **R1** to *y*_0_ = 0.49 for **R3**, indicating an almost perfect superposition of quinoidal and diradical resonance as the best description for the longest molecular wire considered here. The synthesis and single-molecule characterization of **R1**–**R3** are described in detail in our previous study.^21^

To fabricate SAMs, we incubated template-stripped Au (Au^TS^) electrodes in toluene solutions saturated with the respective **R1**–**R3** molecules for 24 h, followed by rinsing with anhydrous toluene and drying under a nitrogen stream. The use of the ultraflat Au^TS^ substrate effectively suppresses leakage currents arising from defect sites associated with surface roughness, thereby enabling reliable characterization of molecular transport properties. Details of the AFM scratch measurements are provided in the SI. The AFM scratch measurements^29–31^ revealed the formation of molecular films with thicknesses of 1.70 ± 0.22, 1.87 ± 0.24, and 2.10 ± 0.22 nm for **R1**, **R2**, and **R3** SAMs, respectively (Figure S4 in the SI). The overall trend in the measured film thicknesses is consistent with the molecular dimensions estimated from the reported X-ray diffraction (XRD) structures and DFT calculations (Table S5), supporting the successful formation of the desired SAMs. For the following analyses of large-area junctions, we use the thickness determined using the AFM scratch measurement as a tunneling distance (*d*), as it more directly reflects the effective thickness of the molecular layers in the junctions.

To further confirm the formation of the SAMs and examine their chemical composition, we conducted X-ray photoelectron spectroscopy (XPS). The corresponding XPS spectra are displayed in Figures S7–S9 in the SI. The high-resolution S 2p spectra of the **R1**–**R3** SAMs on Au^TS^ were fitted with two spin–orbit doublets corresponding to two distinct sulfur environments. The Au–S doublet appears at 161.9–162.1 eV (S 2p_3/2_) and 163.1–163.3 eV (S 2p_1/2_), whereas the molecular C–S doublet appears at 163.3–163.5 eV (S 2p_3/2_) and 164.5–164.7 eV (S 2p_1/2_).^32^ The Au–S signals observed for all three SAMs support successful adsorption of **R1**–**R3** on Au^TS^. The C 1s high-resolution spectra were deconvoluted into components at 284.5–285.0, 286.0–286.3, and 288.8–289.0 eV, corresponding to carbon in C–C/C=C/C≡C bonds, C–X (X = S, O) bonds, and oxidized adventitious carbon species including O–C=O, respectively. The adsorption of adventitious species may be facilitated by substantial intermolecular free volume arising from the sterically bulky molecular structures. For **R2** and **R3** SAMs on Au^TS^, distinct Si 2p signals from the TIPS groups were not resolved, likely due to overlap with the broad Au 5s feature. To verify the presence of intact TIPS groups, we performed high-resolution XPS measurements of **R2** and **R3** SAMs on Ag^TS^ (Figures S10 and S11). In addition to C 1s and S 2p signals, distinct Si 2p_1/2_ and 2p_3/2_ peaks were observed at 102.8 and 102.1 eV, respectively. These results support the retention of the TIPS groups upon surface deposition. Together with the AFM thicknesses, these results indicate the successful formation of **R1**–**R3** molecular layers with relatively extended molecular configurations on Au^TS^.

The **R1**–**R3** molecules have been reported to exhibit an anomalous tunneling behavior at the single-molecule level,^21^ where the tunneling current increases with molecular length due to the progressive stabilization of the diradical character. To investigate whether this diradical character is transferable to the large-area molecular junctions, we measured the current density (*J*) using eutectic gallium-indium (EGaIn)-based junctions (see the experimental methods for detailed protocols). All log tunneling current density histograms displayed a normal distribution (Figure S5 in the SI). Figure 3a shows the *J*–*V* curves of **R1**–**R3** large-area junctions. The tunneling current follows the simplified Simmons tunneling model, *J* = *J*_0_ e^–*d*β^, where *d* is the tunneling distance, *J*_0_ the extrapolated zero-length current density, and β the attenuation coefficient. The **R1**–**R3** large-area junctions exhibited a negative β value of −3.20 ± 1.29 nm^–1^ (Figure 3b), meaning that the conductance increases with the length. While the magnitude of β is less than that of single-molecule junctions (−6.1 nm^–1^) and calculated β in Figure 2c (−16.6 nm^–1^), likely due to intermolecular interactions and electrostatic effects, the negative attenuation behavior is remarkably preserved upon scaling to large-area molecular junctions.

**Figure 3:**
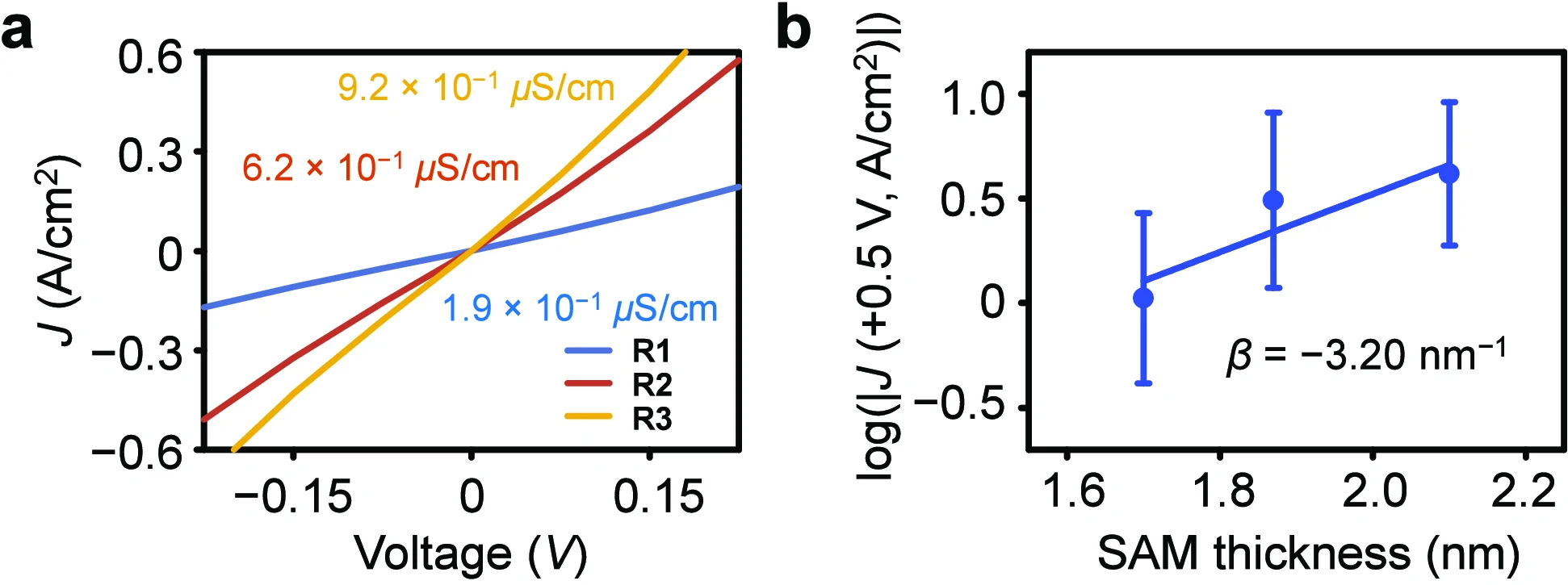
Experimentally measured conductance of bis­(indeno)­acene diradicaloids. (a) Magnified view of the tunneling current density (*J*)–voltage (*V*) curves obtained from the **R1**–**R3** large-area junctions near zero bias. The full *J*–*V* curves on linear and semilogarithmic scales are provided in Figure S6 in the SI. (b) Length dependence of the log |*J* (+0.5 V)| obtained from **R1**–**R3** large-area junctions.

To *S* values of the **R1**–**R3** SAMs were determined in the same junctions (see the Experimental Methods section for detailed experimental procedures).^33–38^ Figure 4a–c shows the thermovoltage (Δ*V*) histograms collected from the **R1**–**R3** large-area junctions under three different temperature differences (Δ*T* = 4, 8, and 12 K). All histograms were fitted with a Gaussian distribution to extract the mean (Δ*V*_mean_) and standard deviation (σ_Δ*V*_) values. All fitted parameters are summarized in Table S1 of the SI. By linearly fitting the Δ*V*_mean_–Δ*T* plots (Figure 4d), *S* values were determined to be 17.5 ± 0.1, 30.4 ± 3.2, and 40.0 ± 1.2 μV/K for **R1**, **R2**, and **R3**, respectively. The positive sign of these *S* values indicates that the transport is HOMO dominated in **R1**–**R3** SAMs. The increasing trend of *S* with molecular length is in good qualitative agreement with our theoretical predictions (Figure 2d and Figure 4e). The *S* values of **R2** and **R3** are higher than the best-performing Blatter radical (*S* = 25.9 μV/K and *G* = 10^–3.5^ *G*_0_) reported previously,^17^ whereas **R1** is lower. This demonstrates that increasing the length of radicals can be used as an effective strategy for the systematic enhancement of *G* and *S*, and, consequently, the power factor.

**Figure 4:**
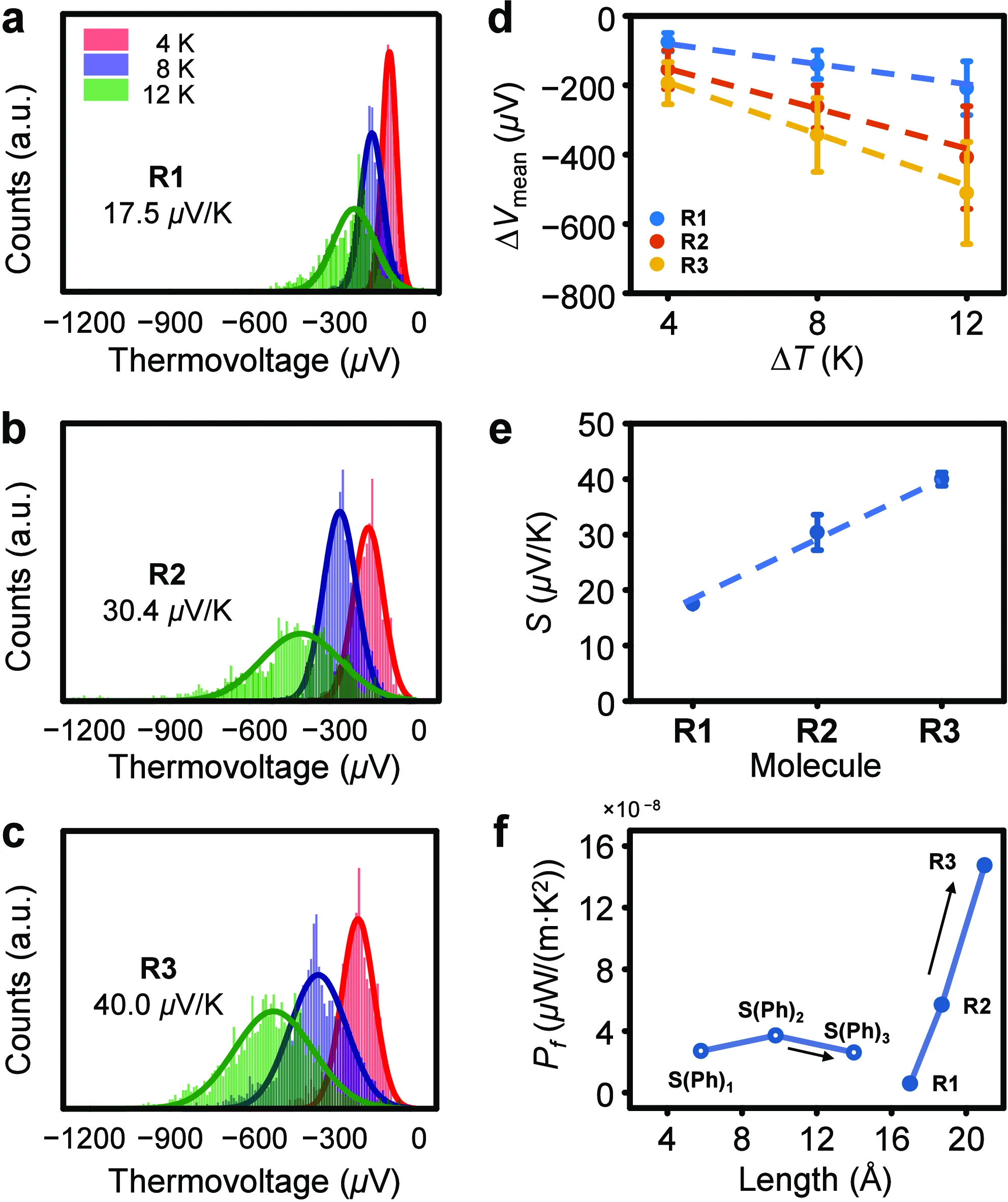
Experimentally measured thermoelectric properties of bis­(indeno)­acene diradicaloids. (a–c) Thermovoltage histograms of **R1**–**R3** large-area junctions collected under three distinct temperature differences (Δ*T* = 4, 8, 12 K). (d) Plots of the mean thermovoltage (Δ*V*_mean_) as a function of Δ*T*, and the extracted Seebeck coefficient (*S*) from each Δ*V*_mean_–Δ*T* plot. (e) Length dependence of the *S* values measured using the EGaIn-based junction technique. (f) Comparison of the thermoelectric power factor (*P*_f_) of the **R1**–**R3** large-area junctions and benchmarking oligophenylene thiols (S­(Ph)_*n*_) as a function of molecular length. The reference values for S­(Ph)_*n*_ are adopted from ref 39.

The length dependence of *S* is often described by the simplified semiempirical equation: *S* = *d* · β_S_ + *S*_0_. Here, *d* is the tunneling barrier width (defined as the film thickness of the **R1**–**R3** SAMs measured using the AFM-scratch method), *S*_0_ is the hypothetical extrapolated *S* at *d* = 0, and β_S_ is an empirical length dependence factor. Conjugated molecular wires typically exhibit positive β_S_ because the HOMO–LUMO gap decreases with increasing conjugation length. For conventional conjugated molecules, such as oligophenylene (di)­thiols,^33,40,41^ heteroacenes,^42^ oligothiophenes^43^ and imine-condensed molecular wires,^44^ β_S_ remains below 10 μV K^–1^ nm^–1^. In contrast, the **R1**–**R3** molecules exhibit a significantly larger β_S_ value of 56.7 ± 3.10 μV K^–1^ nm^–1^. This enhanced length dependence implies a more pronounced reduction in the HOMO–LUMO gap with molecular extension, in agreement with our calculations (Figure 2a).

The concurrent enhancement of both the Seebeck coefficient and current with molecular length leads to a synergistic length dependence in the power factor (*P*_f_ = σ*S* ^2^). To calculate *P*_f_, we obtained the tunneling electrical conductivity (σ) from the slopes of the *J*–*V* curves at *V* = 0 V for the **R1**–**R3** large-area junctions^39,45^ (detailed methods for the evaluation of σ and *P*_f_ are described in the Experimental Methods section). The σ values of the **R1**, **R2**, and **R3** are determined to be 1.9 × 10^–1^, 6.2 × 10^–1^, and 9.2 × 10^–1^ μS cm^–1^, respectively, reflecting the negative tunneling attenuation of the current density. Figure 4f compares the length dependence of *P*_f_ between the **R1**–**R3** large-area junctions and oligophenylene thiols (S­(Ph)_*n*_), a standard conjugated system in molecular thermoelectrics. In conventional molecular junctions, the exponential decay of conductance with increasing molecular length generally outweighs any enhancement in *S*, resulting in a bell-shaped *P*_f_ dependence that typically reaches a maximum at short lengths (e.g., *n* = 2) and subsequently decreases upon further molecular elongation.^39,46^ In contrast, the **R1**–**R3** series exhibits a monotonic increase in *P*_f_ with molecular length. Consequently, the **R3** yields a 4.0-fold enhancement in the power factor compared to the S­(Ph)_2_ reference. We note that while an increased power factor with diradicaloid length promises higher thermoelectric performance, fully quantifying device efficiency and *ZT* requires measuring total thermal conductance—even though it is typically low (<30 pW/K (ref 11)) in organic molecules.

In summary, we have demonstrated that diradicaloid molecular wires offer a robust pathway to high-performance organic thermoelectrics. By engineering a sterically protected open-shell electronic configuration, the **R1**–**R3** series exhibits a rapidly reducing HOMO–LUMO gap with increasing molecular length. This aligns sharp transport resonances closely with the Fermi level as demonstrated by our calculations, overcoming the standard molecular thermoelectric tradeoff to simultaneously enhance electrical conductance and the Seebeck coefficient. Experimental realization via SAM devices confirmed these predictions, demonstrating that the unique quantum transport and orbital characteristics of single diradical molecules translate effectively to scalable, technologically relevant large-area monolayer junctions. This structural paradigm holds exceptional promise for next-generation energy harvesting technologies.

## Computational Methods

We optimized the geometries of **R1**–**R3** using the SIESTA^23^ implementation of density functional theory (DFT), with a double-ζ polarized basis set (DZP). The molecules were relaxed to get the optimized ground state geometry using the Local Spin Density Approximation (LSDA) of the exchange and correlation functional and Ceperley–Alder (CA) parametrization. The geometry was relaxed to a force of 0.01 eV/Å. The transmission coefficient *T*(*E*), as implemented in the GOLLUM^24,25^ transport code for electrons of energy *E* (passing from the source to the drain) is expressed as *T*(*E*) = Tr­(Γ_L_^*e*^(*E*)*G*_*e*_^R^(*E*) Γ_R_^*e*^(*E*)*G*_*e*_^R^†^^)­(*E*)) where Γ_L_^*e*^(*E*) = *i*(Σ_L,R_^*e*^(*E*) – Σ_L,R_^*e* ^†^^(*E*)) describes the level broadening due to coupling between the left and right electrodes (denoted by L and R, respectively) and the central scattering region. Γ_L,R_^*e*^(*E*) is the retarded self-energy associated with the coupling and *G*_*e*_^R^ (*E*) = (*ES* – *H* – Σ_L_^*e*^ – Σ_R_^*e*^)^−1^ is the retarded Green’s function. *H* is the Hamiltonian, and *S* is the overlap matrix obtained from SIESTA implementation of DFT. The DFT+Σ approach has been employed for spectral adjustment.^16^ For this, we used the experimentally measured optical HOMO–LUMO gaps (*E*_g_) of **R1**–**R3** (Table S4) to determine the frontier-level separation, rather than relying on a purely computational estimate of this separation (Table S6), as commonly employed in DFT-based corrections.^47^ We then included the effect of the image charge (*Z*) by shifting the occupied levels upward and the unoccupied levels downward in energy by *Z* as explained in ref 24. This reduces the gap size to *E*_g_ – 2*Z*. The corrected position of the HOMO resonance energy is then given by *E*_*H*_^′^ = *E*_*H*_ + *Z* – *E*_*F*_^Au^, where *E*_*H*_^′^, *E*_*H*_, *Z*, and *E*_*F*_^Au^ are the new position of the HOMO, the Kohn–Sham HOMO position of the isolated molecule calculated from DFT, the image charge, and the Fermi energy of gold, respectively (see Table S6 of the SI for details). The LUMO and higher unoccupied levels are then positioned relative to the corrected HOMO using the experimentally determined gap and the corresponding image-charge correction. Figure S12 shows the DFT transmission functions obtained before and after the Σ corrections.

### Thermoelectric Calculations

Using the approach explained in ref 16, the electrical conductance *G* (*G* = *G*_0_ *L*_0_) and the Seebeck coefficient *S* (*S* = −*L*_1_/*eTL*_0_) are calculated from the electron transmission coefficient *T*(*E*), where *L*_*n*_ = ∫_–∞_^+∞^ d*E* (*E* – *E*_F_)^*n*^ *T*(*E*)­(−∂*f*(*E*,*T*,*E*_F_)/∂*E*) and *f* is the Fermi–Dirac probability distribution function *f* = (e^(*E*–*E*_F_)/*k*_B_ *T*^ + 1)^−1^, *T* is the temperature, *E*_F_ is the Fermi energy, *G*_0_ = 2*e* ^2^/*h* is the conductance quantum, *e* is the electron charge, and *h* is the Planck’s constant.

## Experimental Methods

### SAM Formation and Characterization

Freshly prepared substrates were immersed overnight in saturated solutions of **R1**–**R3** in anhydrous toluene, which were filtered through a 0.2 μm PTFE syringe filter before use. The samples were then removed from the solution, thoroughly rinsed with pure toluene, and dried under a N_2_ flow. All solvents were degassed by N_2_ bubbling and three freeze–pump–thaw cycles, and all transfer steps were performed in a N_2_-filled glovebox (O_2_ < 0.1%). The AFM scratch method was used to estimate the film thickness, following protocols reported in the literature and in our previous work.^29–31^ AFM measurements were performed using a Multimode 8 system (Bruker). Briefly, a 1 μm × 1 μm area of the film was removed by scratching in contact mode using SCANASYST-AIR probes with a resonant frequency of 70 kHz and a spring constant of 0.4 N/m. The peak force set point was set to 4 V during scratching. Subsequently, topographic images were acquired in ScanAsyst Air mode over a larger 5 μm × 5 μm region. During imaging, the peak force set point was automatically adjusted by feedback control to prevent damage to the remaining film. The XPS measurements were conducted using Thermo Scientific K-Alpha photoelectron spectrometer equipped with a monochromated Al Kα source (1486.6 eV). The binding energies were calibrated by setting the Au 4f_7/2_ peak of the Au^TS^ to 84.0 eV and the Ag 3d_5/2_ peak of the Ag^TS^ to 368.2 eV. Peak fitting was performed using XPS Peak Fit software.

### Thermoelectric Measurement

Thermoelectric measurements were conducted following established protocols reported in our previous studies.^33–35,37,38^ All samples were positioned on the Peltier devices, which heat the substrate to induce a temperature gradient (Δ*T*) across the molecular junctions. Significant heat-carrier scattering at the metal/molecule and molecule/metal interfaces facilitated the formation of Δ*T*.^33,34,48^ To quantify the Δ*T*, the temperatures of both the substrate and the ambient were monitored during the measurements. Conical EGaIn electrodes were gently brought into contact with the diradicaloid molecular films to form large-area molecular junctions. The geometric contact areas of the junctions were confirmed via 480× magnification optical microscopy, assuming a circular contact geometry, and consistently maintained at approximately 1.9 × 10^9^ nm^2^. To ensure reproducibility and eliminate potential contamination, conical EGaIn electrodes were freshly fabricated prior to each junction formation. Each junction was monitored for 30 s, yielding 76 data points per junction. Δ*V* data were collected to construct histograms, and each histogram shows single Gaussian distributions. The mean (Δ*V*_mean_) and standard deviation (σ_Δ*V*_) values extracted from these distributions were used to construct Δ*V*_mean_–Δ*T* plots. Linear fitting of the Δ*V*_mean_–Δ*T* data enabled the determination of the Seebeck coefficient (*S*, μV/K) for the large-area molecular junctions.

### Tunneling Current Measurement

Electrical characterization of the same junctions used for thermopower measurements was performed in accordance with previously reported methods.^45^ All measurements were conducted under ambient conditions. Each *J*–*V* trace consisted of a voltage sweep sequence as follows: 0 V → +0.5 V → 0 V → −0.5 V → 0 V, corresponding to two complete scans per trace. The contact area diameter was determined at high magnification (480×), assuming a circular geometry, and this value was used to calculate the current density (*J*, A/cm^2^). The yield (%) of operational junctions was calculated as the ratio of functional junctions to the total number of junctions tested, excluding those that short-circuited. Shorted junctions, which were identified by currents reaching the compliance limit of the electrometer (±0.105 A in this study), were excluded from further analysis as they do not reflect the properties of the diradicaloid molecular films and can skew current density distributions toward higher values. Distributions of log-current density (log |*J*|) were analyzed by fitting to a Gaussian distribution, yielding mean (μ_*J*(*V*)_) and standard deviation (σ_*J*__(*V*)_) values at a given voltage (*V*). The extracted values are summarized in Table S2.

### Evaluation of Electrical Conductivity and Power Factor

For the large-area junctions, the electrical response is expressed by an effective tunneling conductivity (σ), obtained from the measured current density (*J*) and electric field (*E* = *V*/*d*).^45^ As *S* is defined under zero-bias conditions, extracting σ from the *V* = 0 V ensures consistency in evaluating the power factor (*P*_f_). Thus, σ was obtained from the slope of *J*–*V* curves at *V* = 0 V (Figure 3a), using^39,45^ σ = *J*/*E* = *d*·(d*J*/d*V*)_*V*=0_ (μS cm^–1^), where *E* is the electric field, defined as *E* = *V*/*d*. Here, the electrode spacing (*d*) was estimated from the film thickness of **R1**–**R3** SAMs measured using AFM scratch method. The *P*_f_ parameter was calculated as *P*_f_ = σ · *S* ^2^ (μW m^–1^ K^–2^). The *P*_f_ values were further corrected by considering the ratio of the effective contact area of the EGaIn tip to its geometrical contact area (10^–3^–10^–6^), as reported in previous studies.^45^ The calculated values of σ and corrected *P*_f_ are summarized in Table S3.

## Supplementary Material



## Notes

Additional details, including further control experiments and additional DFT modeling (PDF)
