# Supplementary material for: Scaling Diradicaloid Thermoelectricity
Source: Nano Lett. 2026 Sep 25;26(39):13635–41. doi: 10.1021/acs.nanolett.6c03798 (PMC13651621; doi:10.1021/acs.nanolett.6c03798)
Supplement: Supplementary file 1 [file nl6c03798_si_001.pdf]

# Supplementary Information

# Scaling Diradicaloid Thermoelectricity

Lewis Hamilton[1,#], Jiung Jang[2,#], Amit Sil[3,4,#,*], Peng He[2], Abdalghani H.S. Daaoub[1], Yongjun Choi[2], Sara Sangtarash[1], Andrea Vezzoli[3], Hyo Jae Yoon[2,*] and Hatef Sadeghi[1,*]

1 Quantum Device Modelling Group, School of Engineering, University of Warwick, Coventry CV4 7AL, UK
2 Department of Chemistry, Korea University, Seoul 02841, Republic of Korea
3 Department of Chemistry, University of Liverpool, Crown Street, Liverpool L69 7ZD, UK
4 Department of Chemical Sciences, Bose Institute, Kolkata, West Bengal 700091, India

# These authors contributed equally to this study
* Corresponding authors: amit.sil@liverpool.ac.uk; hyoon@korea.ac.kr; Hatef.Sadeghi@warwick.ac.uk

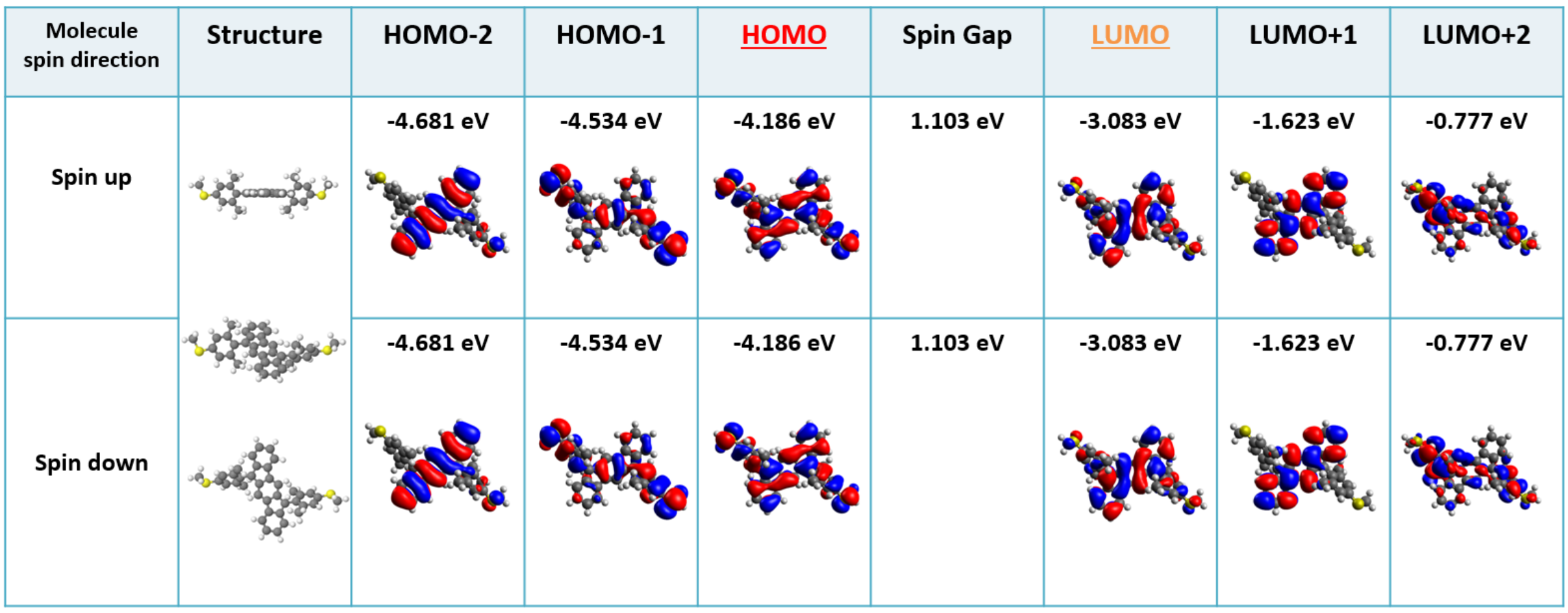

| Molecule spin direction | Structure | HOMO-2 | HOMO-1 | HOMO | Spin Gap | LUMO | LUMO+1 | LUMO+2 |
|---|---|---|---|---|---|---|---|---|
| Spin up | | -4.681 eV | -4.534 eV | -4.186 eV | 1.103 eV | -3.083 eV | -1.623 eV | -0.777 eV |
| Spin down | | -4.681 eV | -4.534 eV | -4.186 eV | 1.103 eV | -3.083 eV | -1.623 eV | -0.777 eV |

Figure S1: Molecular orbital of **R1** calculated using the method explained in the methodology section in the maintext.

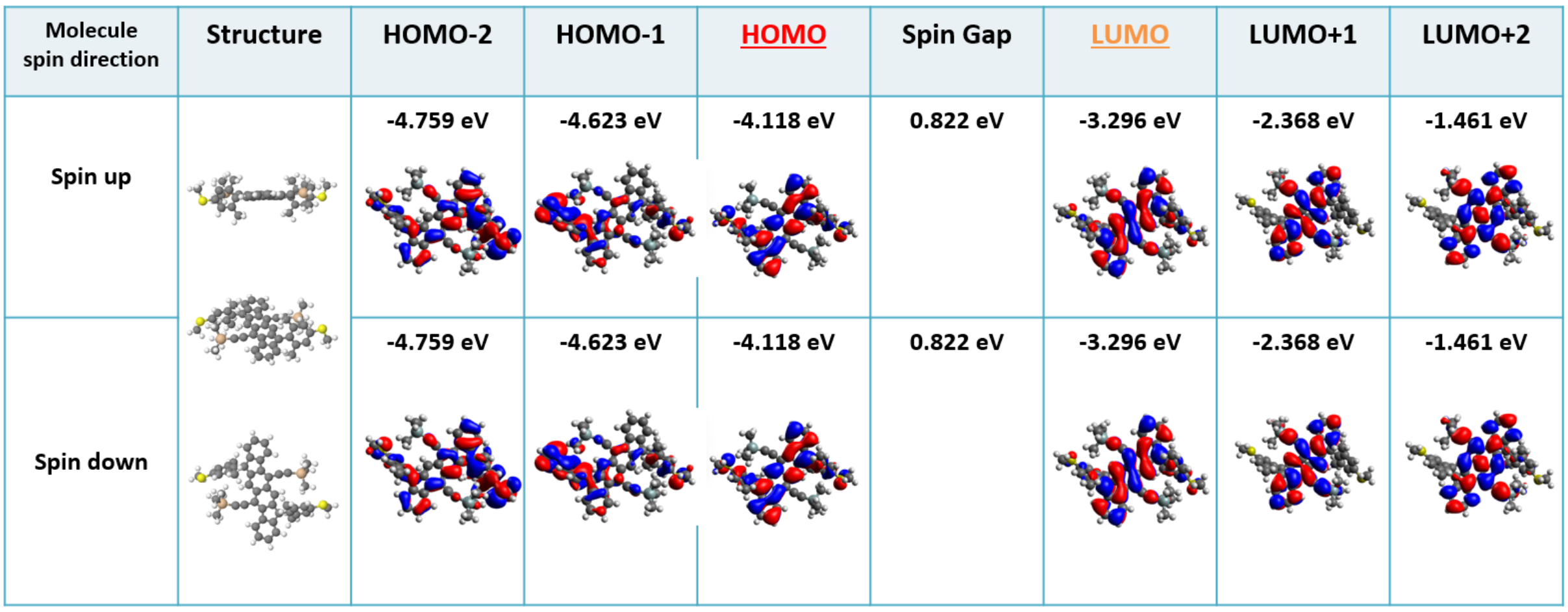

| Molecule spin direction | Structure | HOMO-2 | HOMO-1 | HOMO | Spin Gap | LUMO | LUMO+1 | LUMO+2 |
|---|---|---|---|---|---|---|---|---|
| Spin up | | -4.759 eV | -4.623 eV | -4.118 eV | 0.822 eV | -3.296 eV | -2.368 eV | -1.461 eV |
| Spin down | | -4.759 eV | -4.623 eV | -4.118 eV | 0.822 eV | -3.296 eV | -2.368 eV | -1.461 eV |

Figure S2: Molecular orbital of **R2** calculated using the method explained in the methodology section in the maintext.

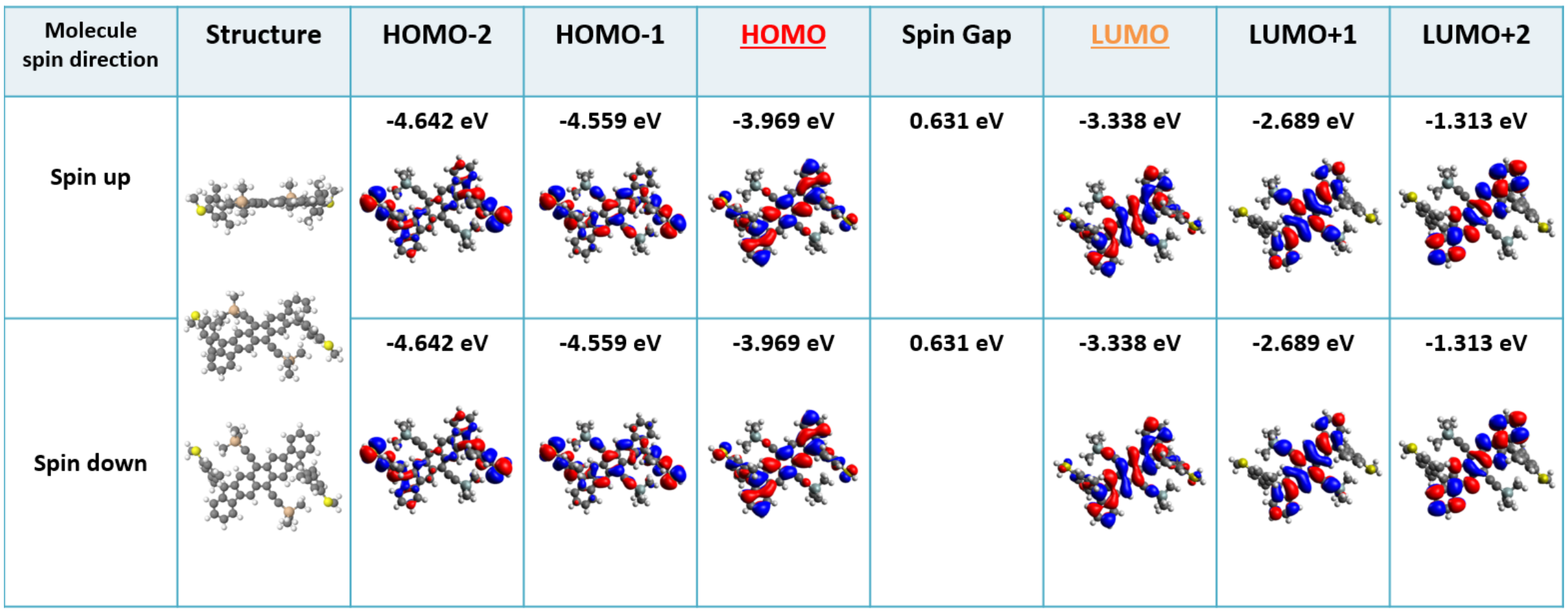

| Molecule spin direction | Structure | HOMO-2 | HOMO-1 | HOMO | Spin Gap | LUMO | LUMO+1 | LUMO+2 |
|---|---|---|---|---|---|---|---|---|
| Spin up | | -4.642 eV | -4.559 eV | -3.969 eV | 0.631 eV | -3.338 eV | -2.689 eV | -1.313 eV |
| Spin down | | -4.642 eV | -4.559 eV | -3.969 eV | 0.631 eV | -3.338 eV | -2.689 eV | -1.313 eV |

Figure S3: Molecular orbital of **R3** calculated using the method explained in the methodology section in the maintext.

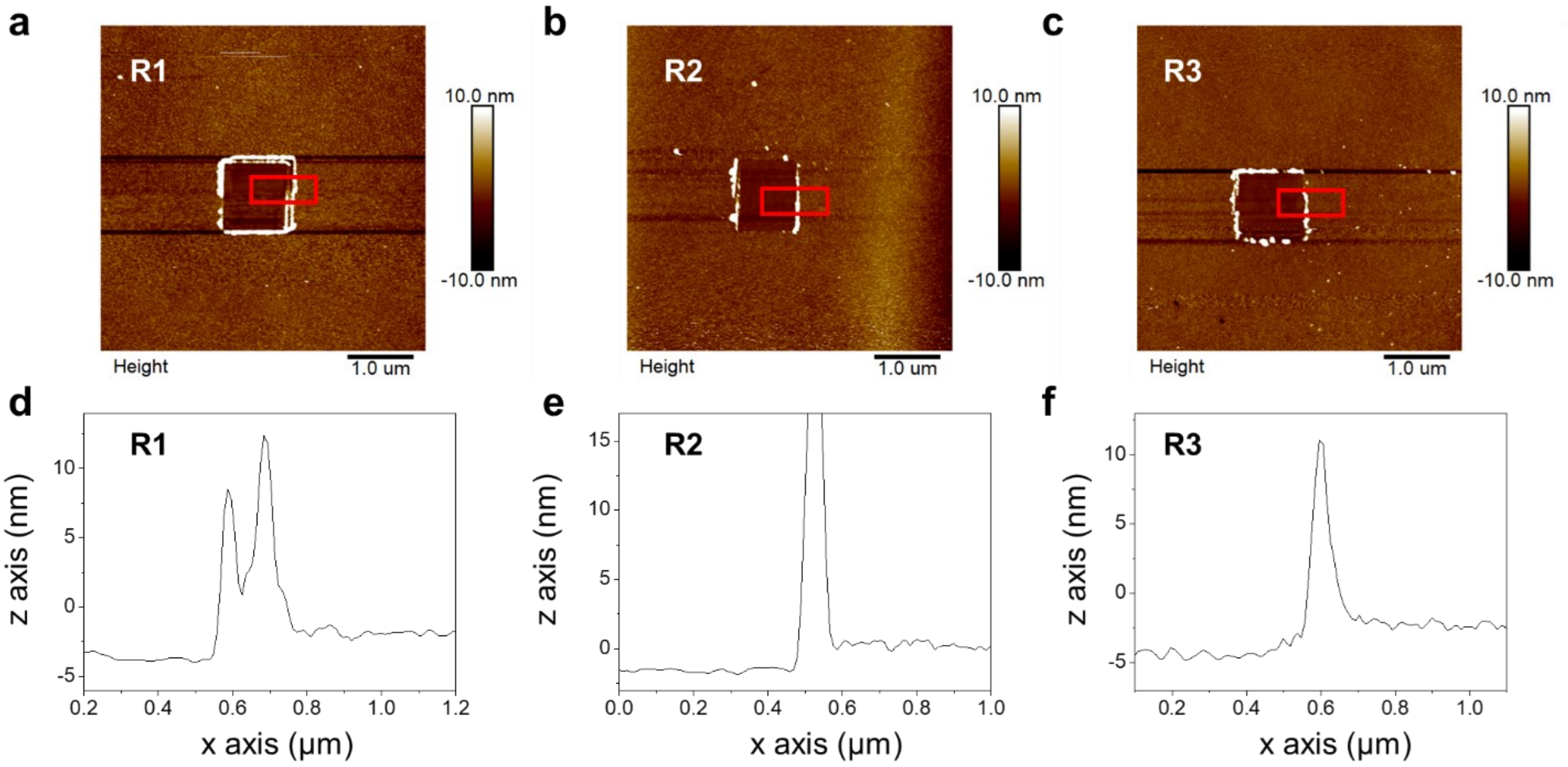


Figure S4: Atomic force microscopy (AFM) topography of molecules (a) **R1,** (b) **R2**, and (c) **R3** SAMs after nano-scratching by AFM probe and (d-f) their height profile. (a-c) Red boxes indicate the regime that the profile plots were derived.

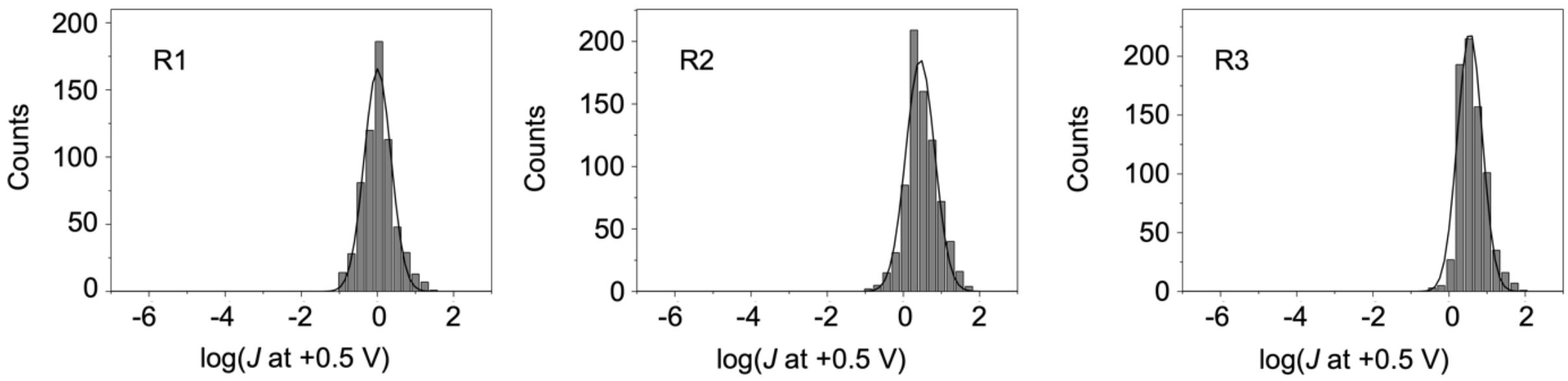


Figure S5: Histograms of log |current density ($J$, A/cm$^2$)| measured on **R1-R3** large-area junctions at +0.5 V. The values of $\mu_J(V)$ and $\sigma_J(V)$ derived from each histogram are summarized in Table S2.

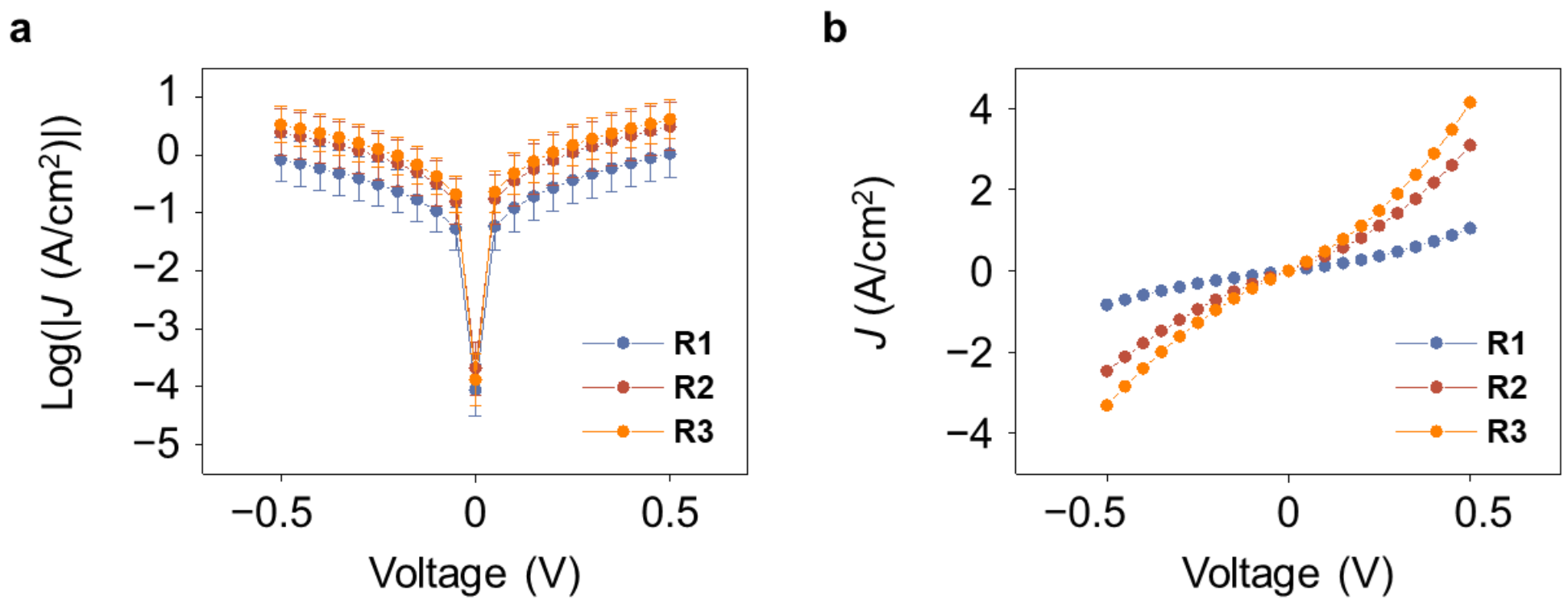


Figure S6: Tunneling current density ($J$)-voltage ($V$) curves obtained from the **R1-R3** large-area junctions over the full measured range from −0.5 to +0.5 V, presented on (a) semilogarithmic and (b) linear $J$ scale.

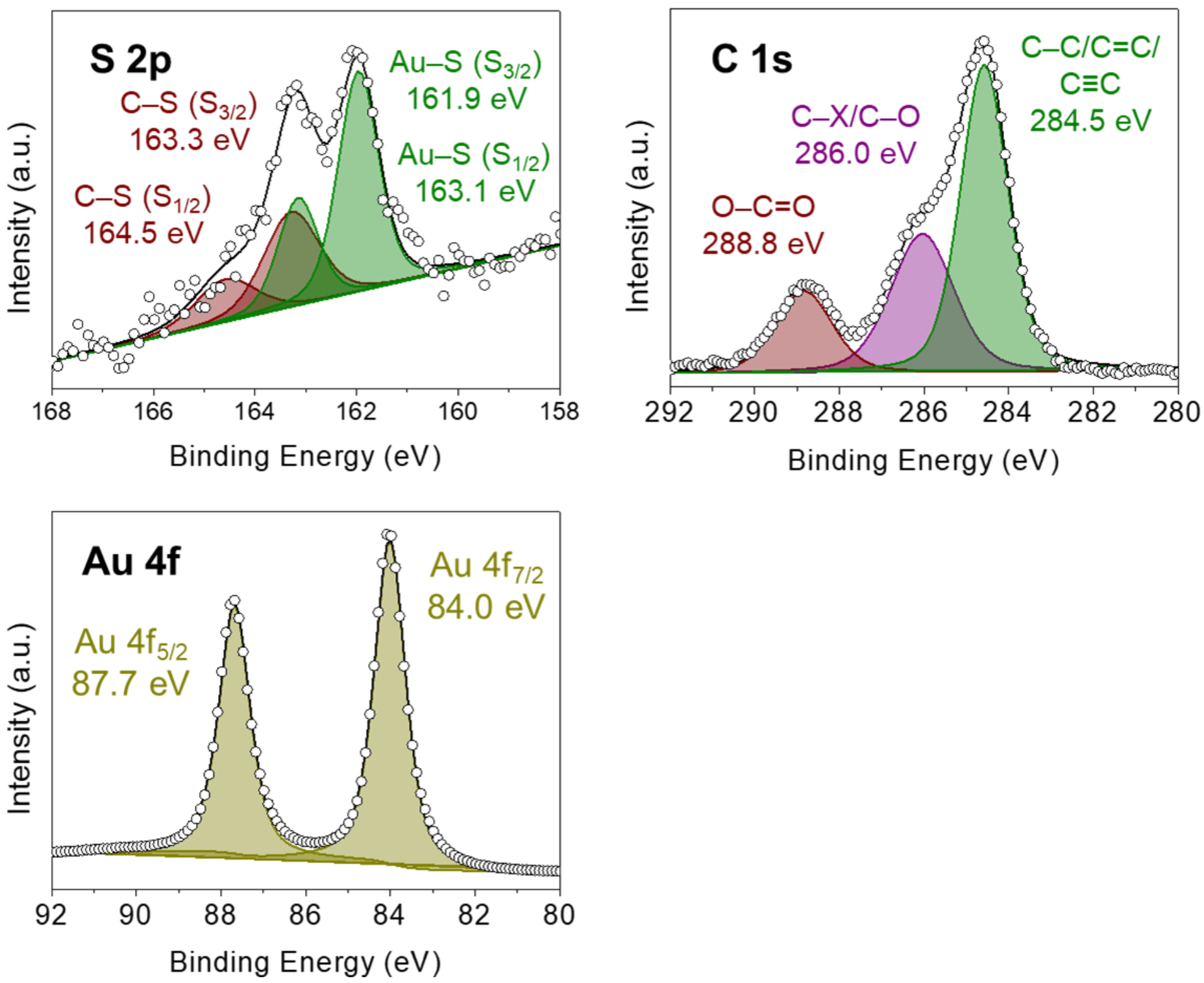


Figure S7: High-resolution XPS spectra of the S 2p, C 1s, and Au 4f regions for **R1** on Au$^{TS}$.

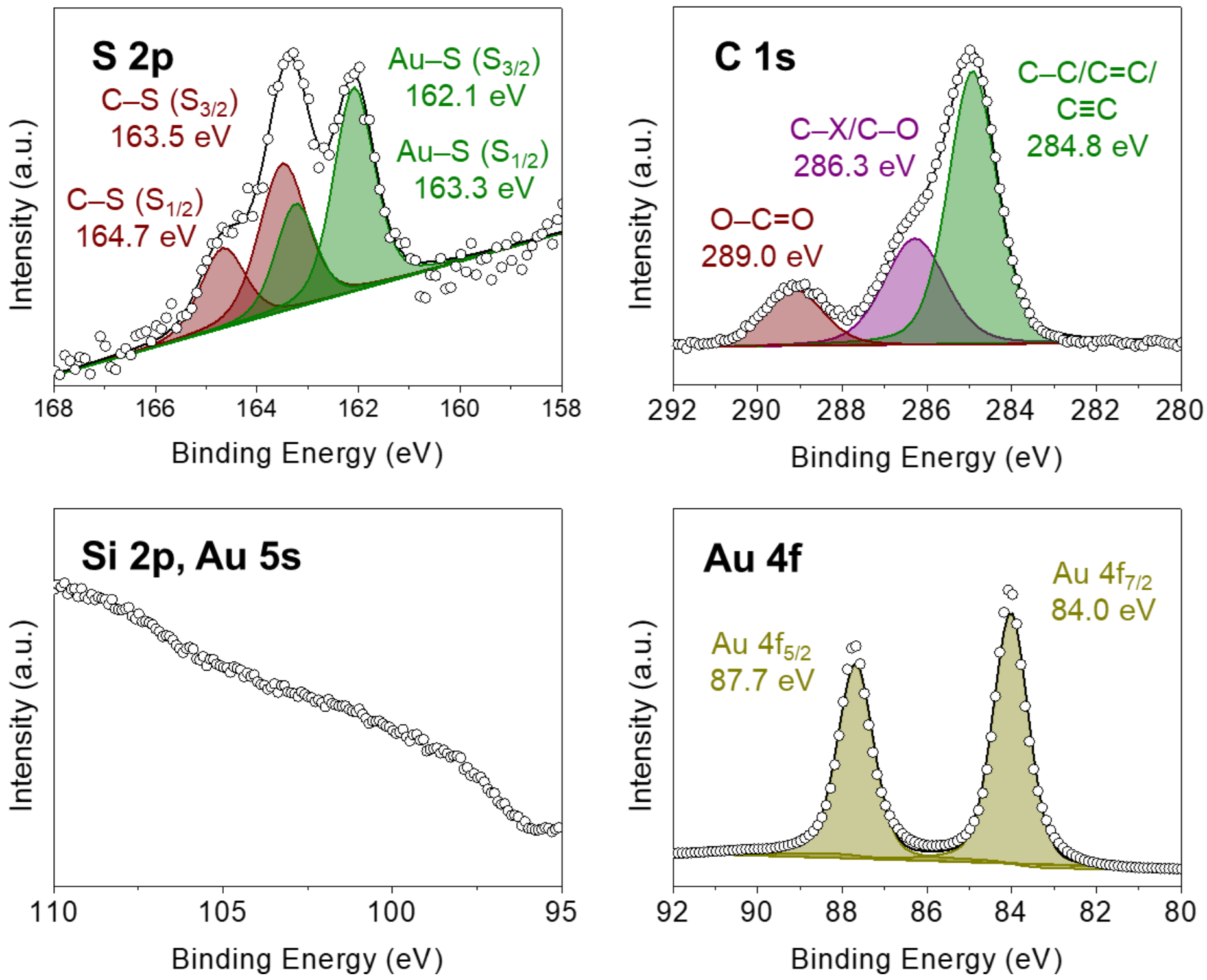


Figure S8: High-resolution XPS spectra of the S 2p, C 1s, Si 2p, and Au 4f regions for **R2** on $Au^{TS}$. The Si 2p region overlaps with the broad Au 5s feature.

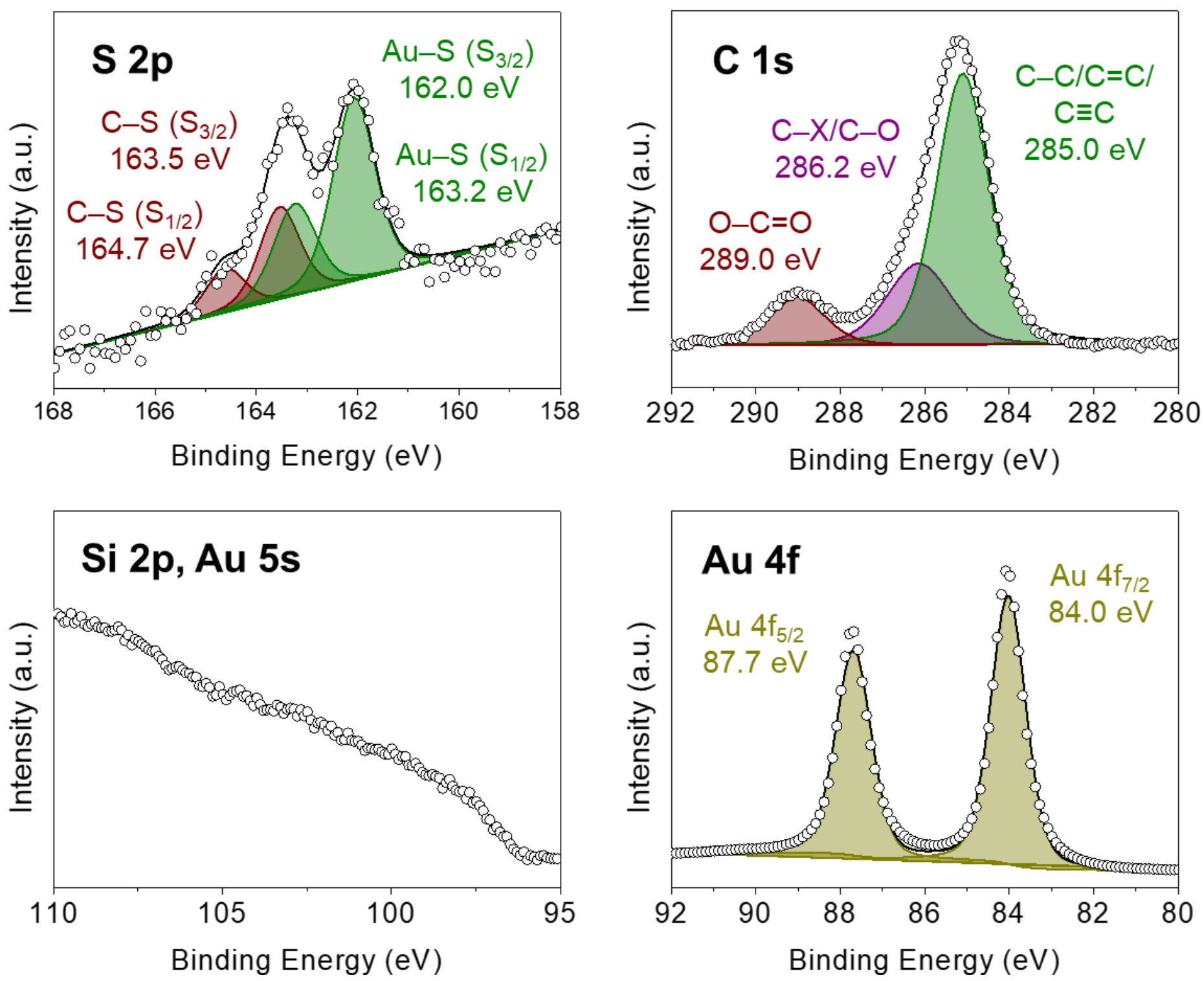


Figure S9: High-resolution XPS spectra of the S 2p, C 1s, Si 2p and Au 4f regions for **R3** on $Au^{TS}$. The Si 2p region overlaps with the broad Au 5s feature.

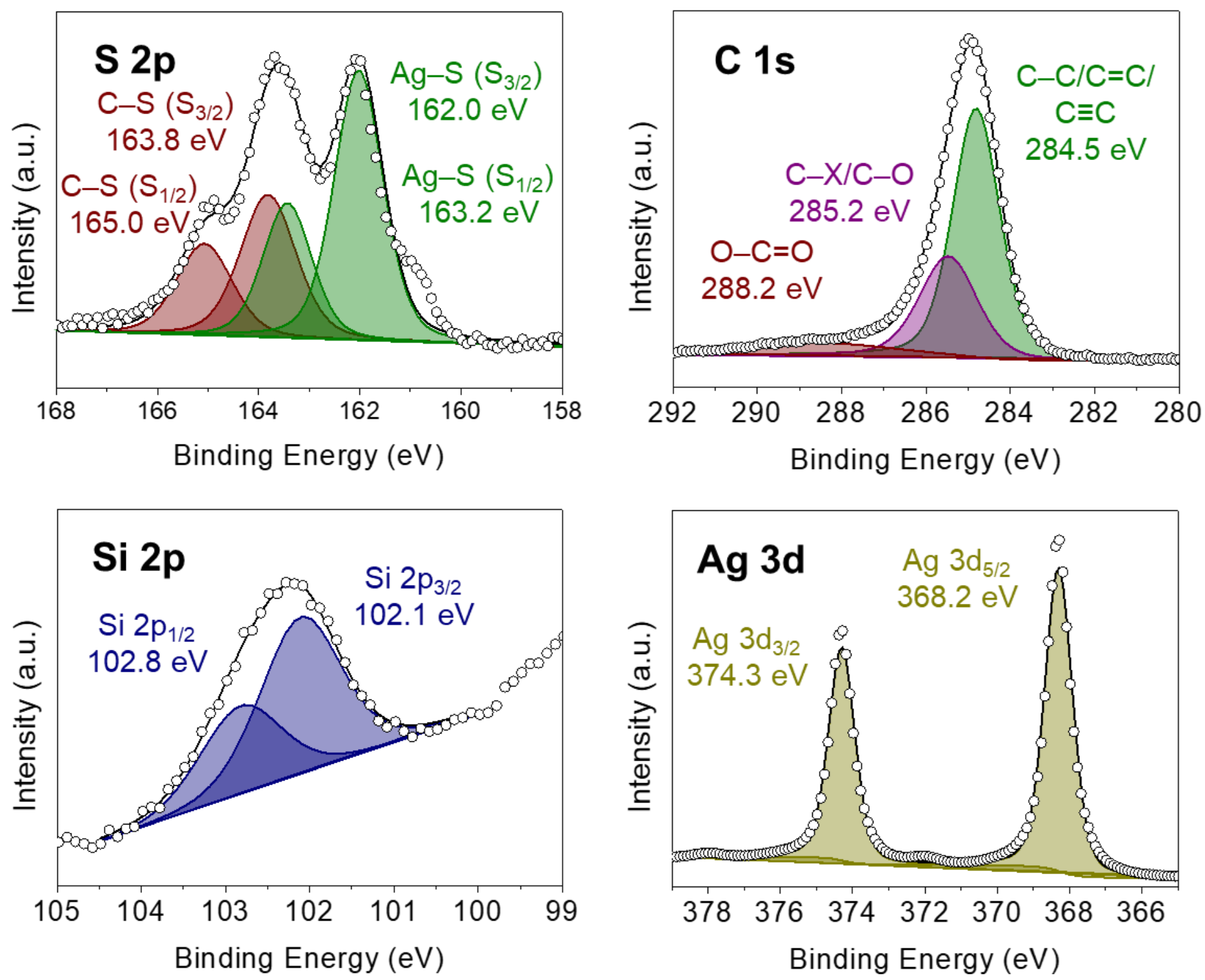

Figure S10: High-resolution XPS spectra of the S 2p, C 1s, Si 2p, and Ag 3d regions for **R2** on $Ag^{TS}$.

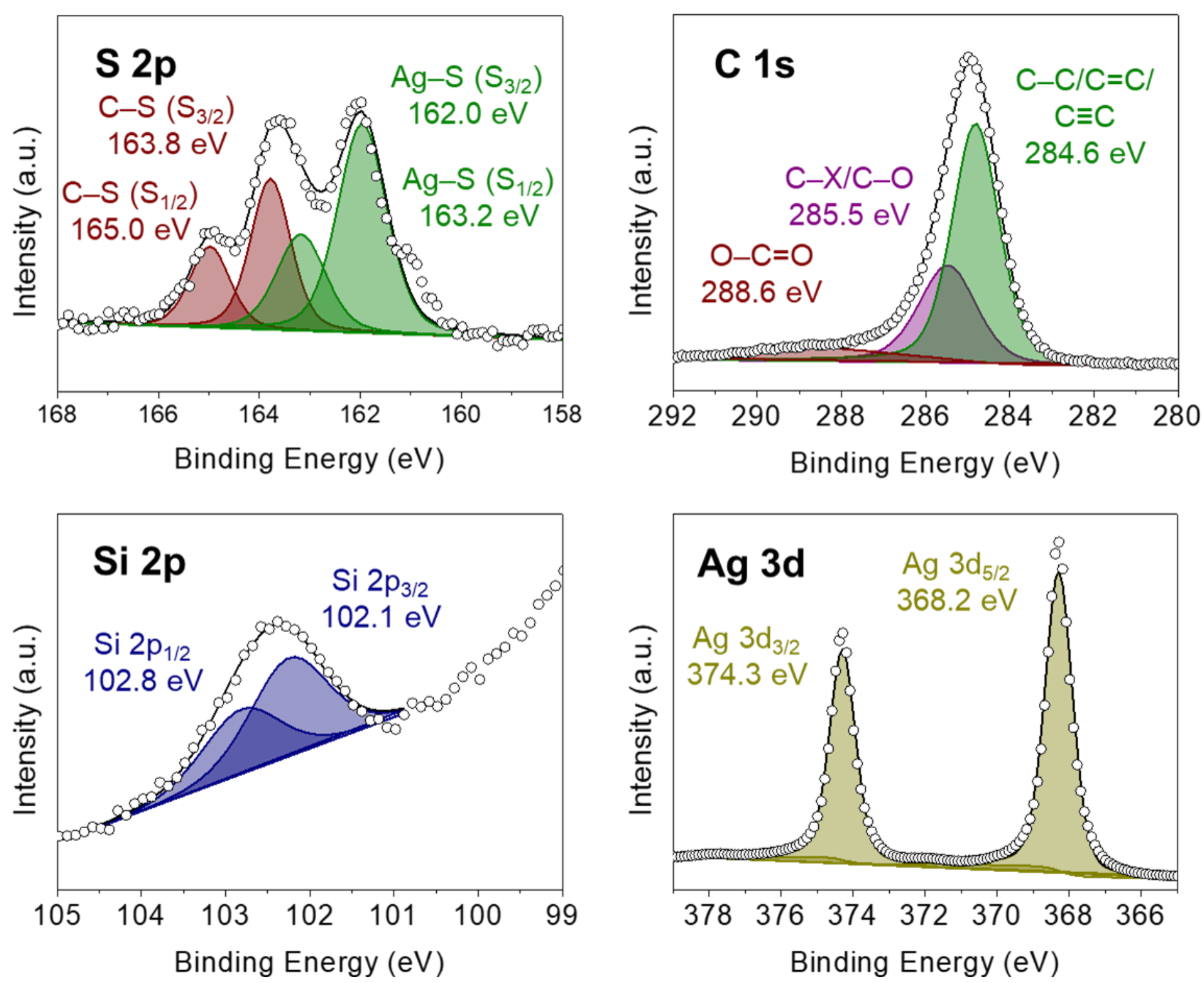

Figure S11: High-resolution XPS spectra of the S 2p, C 1s, Si 2p, and Ag 3d regions for **R3** on $Ag^{TS}$.

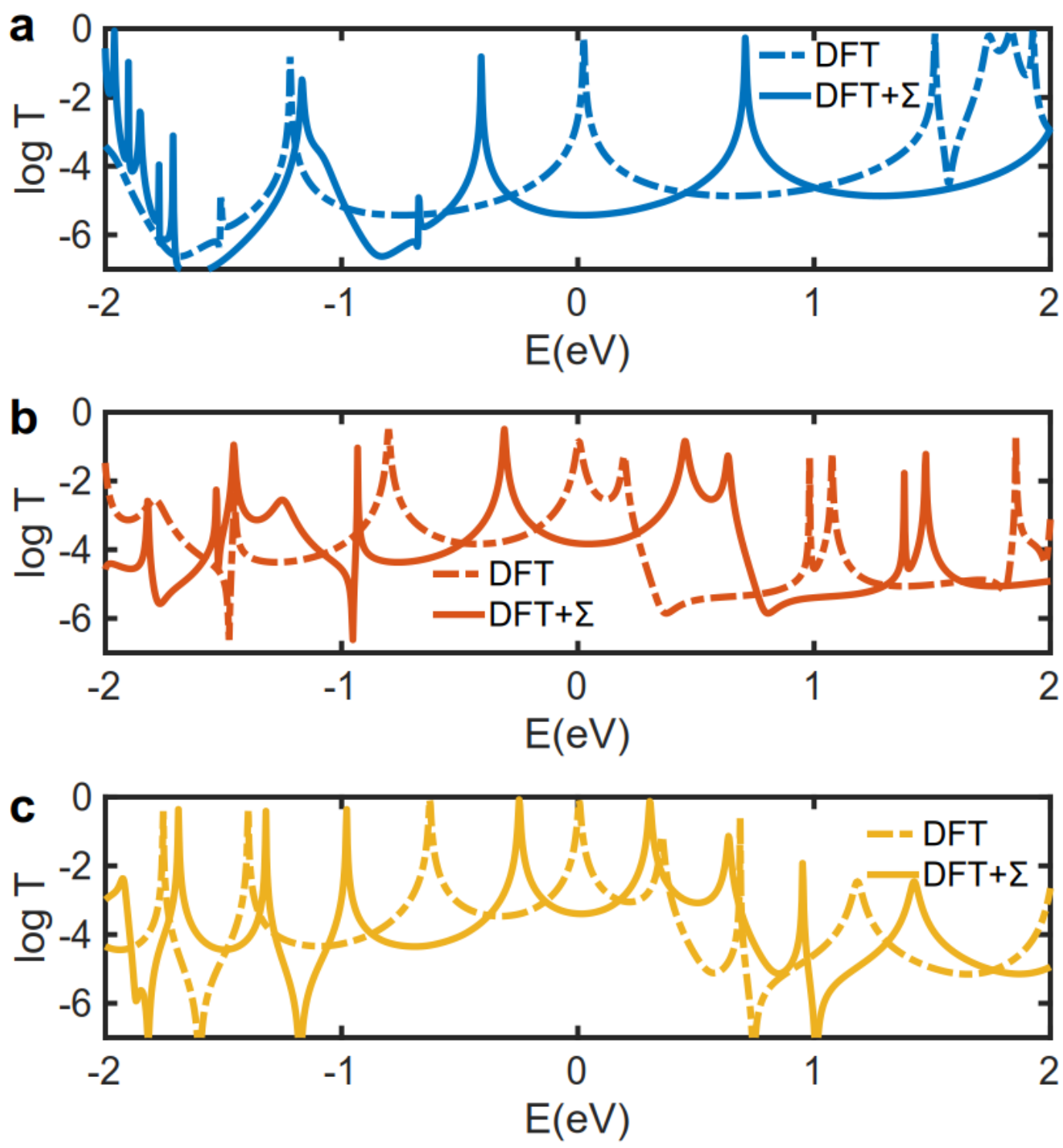


Figure S12: DFT transport calculations with and without the Σ correction for (a) **R1**, (b) **R2**, and (c) **R3**.

Table S1: Summary of thermopower measurements of **R1-R3** molecules. Mean ($\Delta V_{mean}$) and standard deviation ($\sigma_{\Delta V}$) values were estimated by Gaussian curve fitting to thermovoltage ($\Delta V$) histograms in Figure 4a-c.

| **Molecule** | **$\Delta T$ (K)** | **# of junctions** | **Counts** | **$\Delta V_{mean} \pm \sigma_{\Delta V}$ (μV)** | ***S* (μV/K)** | **Yield (%)** |
|---|---|---|---|---|---|---|
| **R1** | 4 | 35 | 2660 | −75.6 ± 26.5 | 17.5 ± 0.1 | 83 |
| | 8 | 35 | 2660 | −141.0 ± 41.2 | | 81 |
| | 12 | 35 | 2660 | −208.4 ± 77.4 | | 78 |
| **R2** | 4 | 40 | 3040 | −154.7 ± 55.9 | 30.4 ± 3.2 | 89 |
| | 8 | 40 | 3040 | −261.5 ± 62.1 | | 89 |
| | 12 | 37 | 2812 | −408.3 ± 148.2 | | 88 |
| **R3** | 4 | 65 | 4940 | −193.9 ± 60.7 | 40.0 ± 1.2 | 93 |
| | 8 | 65 | 4940 | −343.3 ± 106.4 | | 90 |
| | 12 | 64 | 4864 | −510.9 ± 147.3 | | 89 |

Table S2: Summary of *J-V* curve measurements over the **R1-R3** large-area junctions. $N_{junction}$ is the number of junctions measured; $N_{cycle}$ is the number of *J-V* cycles; $\mu_J(V)$ and $\sigma_J(V)$ are mean and standard deviation values of log |*J*| at an external voltage, *V*, respectively, determined from Gaussian curve fitting to the histogram.

| Molecule | $N_{junction}$ | $N_{cycle}$ | $\mu_{J\,(-0.5\,V)} \pm \sigma_J$ | $\mu_{J\,(+0.5\,V)} \pm \sigma_J$ | Yield (%) |
|---|---|---|---|---|---|
| **R1** | 10 | 200 | −0.08 ± 0.39 | 0.02 ± 0.41 | 90 |
| **R2** | 12 | 240 | 0.39 ± 0.40 | 0.49 ± 0.42 | 83 |
| **R3** | 12 | 240 | 0.52 ± 0.31 | 0.62 ± 0.34 | 92 |

Table S3: Summary of tunneling conductivity ($\sigma$), Seebeck coefficient ($S$), and power factor ($P_f$) for **R1-R3** large-area junctions.

| Molecule | $\sigma$ (μS/cm) | $S$ (μV/K) | $P_f$ (μW/(m·K²)) | Corrected $P_f$ [a] (μW/(m·K²)) |
|---|---|---|---|---|
| **R1** | $1.9 \times 10^{-1}$ | 17.5 | $5.8 \times 10^{-9}$ | $5.8 \times 10^{-3} – 5.8 \times 10^{-6}$ |
| **R2** | $6.2 \times 10^{-1}$ | 30.4 | $5.7 \times 10^{-8}$ | $5.7 \times 10^{-2} – 5.7 \times 10^{-5}$ |
| **R3** | $9.2 \times 10^{-1}$ | 40.0 | $1.5 \times 10^{-7}$ | $1.5 \times 10^{-1} – 1.5 \times 10^{-4}$ |

[a] The calculation also considered the effective contact area of the EGaIn tip relative to the geometrical contact area ($10^{-3}$ – $10^{-6}$) reported previously[1,2].

Table S4: Optical bandgap calculated from the UV–Vis spectra, from the edge of the absorption band (by linear fitting of the steepest portion of the edge). We focused on the edge rather than $\lambda_{MAX}$ to reduce the effect of vibronic coupling and structural disorder.

| Molecule | Optical Bandgap (eV) |
|---|---|
| **R1** | 2.19 |
| **R2** | 1.69 |
| **R3** | 1.52 |

Table S5: Comparison of the film thickness of the **R1**-**R3** SAMs determined by AFM with the molecular lengths obtained from XRD structure and DFT calculations (unit: Å).

| Molecule | AFM | XRD | DFT |
|---|---|---|---|
| **R1** | 17.0 | 16.5 | 16.5 |
| **R2** | 18.7 | 17.7 | 17.3 |
| **R3** | 21.0 | 19.5 | 18.3 |

Table S6. The HOMO (H), LUMO (L) and energy gap for molecule **R1**-**R3** including ionization potential (IP), electron affinity (EA), Kohn-Sham (KS) DFT energies for frontier orbitals of isolated molecules, the image charges, and the experimental gap obtained from the UV–Vis spectra measurement. All energies are in eV.

| Molecule | - IP | - EA | IP-EA | KS H | KS L | KS Gap | Exp Gap | Image charge |
|---|---|---|---|---|---|---|---|---|
| **R1** | -4.94 | -2.04 | 2.9 | -4.19 | -3.08 | 1.10 | 2.19 | 0.54 |
| **R2** | -4.91 | -2.16 | 2.75 | -4.12 | -3.30 | 0.82 | 1.69 | 0.53 |
| **R3** | -4.68 | -2.29 | 2.39 | -3.97 | -3.34 | 0.63 | 1.52 | 0.48 |

## References


(1) Simeone, F. C.; Yoon, H. J.; Thuo, M. M.; Barber, J. R.; Smith, B.; Whitesides, G. M. Defining the Value of Injection Current and Effective Electrical Contact Area for EGaIn-Based Molecular Tunneling Junctions. *J. Am. Chem. Soc.* **2013**, *135* (48), 18131–18144.

(2) Chen, X.; Roemer, M.; Yuan, L.; Du, W.; Thompson, D.; Del Barco, E.; Nijhuis, C. A. Molecular Diodes with Rectification Ratios Exceeding 105 Driven by Electrostatic Interactions. *Nat. Nanotechnol.* **2017**, *12* (8), 797–803.
